# Predatory and Parasitic Insects Associated with *Urophora cardui* L. (Diptera: Tephritidae) Galls on Canada Thistle, *Cirsium arvense* L. (Asterales, Asteraceae) in North Dakota

**DOI:** 10.3390/insects13070646

**Published:** 2022-07-18

**Authors:** Stephanie J. Swenson, Jonathan Bell-Clement, Sarah Schroeder, Deirdre A. Prischmann-Voldseth

**Affiliations:** 1 Entomology Department, North Dakota State University, Fargo, ND 58108-6050, USA; stephanie.swenson@uni-kassel.de (S.J.S.); jonbellclement@gmail.com (J.B.-C.); s.schroe17@gmail.com (S.S.); 2 Department of Botany, Institute for Biology, University of Kassel, 34132 Kassel, Germany

**Keywords:** endophage, gall insects, thistle fauna, checkered beetle

## Abstract

**Simple Summary:**

Plant gall-inducing insects can be attacked by a diverse and unique assemblage of predatory and parasitic insects. We surveyed the insect fauna associated with galls induced by the fly *Urophora cardui* L. on Canada thistle, a perennial weed. We found that the thistle gall fly and its primary parasitoid wasp, *Pteromalus elevatus* (Walker), were widespread throughout the study area. In addition, we recovered a checkered beetle, *Isohydnocera tabida* (LeConte), from a subset of galls, which is a new host record and provides vital information on the little-known immatures of this predatory species. This study adds to the taxonomic literature of gall-inhabiting insect species and *I. tabida*.

**Abstract:**

We surveyed the insect fauna associated with *Urophora cardui* L. (Diptera: Tephritidae) galls on Canada thistle, *Cirsium arvense* L. (Asterales, Asteraceae), in parts of the northern Great Plains, U.S., by field-collecting galls and rearing or dissecting out the insects. We also examined the relationships between gall biomass and insect density and biomass. *Urophora cardui* were widespread, and the gall biomass was positively correlated with fly density and fly biomass. We recovered *Isohydnocera tabida* (LeConte) (Coleoptera: Cleridae) from galls in two counties, which represents a new host record and provides vital information on the little-known immatures of this predatory species. *Pteromalus elevatus* (Walker) (Hymenoptera: Pteromalidae) was the dominant parasitoid that emerged from the *U. cardui* galls. Individual galls typically only had one insect species, and occasionally both *U. cardui* and *P. elevatus* were present, but it was rare for other insects to be present in galls housing *I. tabida*. This study adds to the taxonomic literature of gall-inhabiting insect species and provides new information on the predators of *U. cardui*, specifically a little-known clerid beetle species.

## 1. Introduction

*Urophora cardui* L. (Diptera: Tephritidae) is a biological control agent of the noxious weed Canada thistle, *Cirsium arvense* L. (Asterales, Asteraceae) native to Europe, and this insect induces stem galls. Canada thistle is the primary host plant for *U. cardui*, although this insect is associated with two other thistle species, *C. creticum* (Lam.) and *C. setosum* (Willd.) Besser ex M. Bieb. [1,2,3]. A classical biocontrol program against *C. arvense* began in North America in the mid-1900s, and between 1963 and 1996, six insect species were deliberately released or redistributed in order to suppress *C. arvense* populations [4,5]. *Urophora cardui* was first released in Canada in 1974 [6] and in the U.S. in 1977 in California [7]. It has since been released in multiple other states and is now widely distributed within Europe and across the U.S. and southern Canada [7,8,9,10].

Feeding by *U. cardui* can negatively impact Canada thistle growth and fitness, especially in younger stems [8,11]. Adult female *U. cardui* oviposit on shoot tips among developing leaves [12] in June and July [6,11,13]. Females can lay 11 to 14 eggs per clutch [11,14], which hatch after 7 to 10 days [12]. Larvae move into the stem, which triggers gall initiation, followed by gall growth and maturation, with galls becoming woody and hard due to tissue lignification [12,13,15]. Galls have multiple chambers (i.e., are multilocular), with one larva per chamber, and can house up to a dozen individuals that feed on nutritive cells [12,13,16,17,18], with greater larval density resulting in larger galls and increased fly fitness [14]. *Urophora cardui* is univoltine and overwinters as third instar larva within the gall, with pupates emerging in the spring [11,13,14].

Endophagous insects that spend part of their life cycle inside plant tissues are often parasitized by hymenopterans [19]. Several species of wasps, primarily in the Eurytomidae and Pteromalidae families, parasitize *Urophora* within galls [10,17,20,21], and parasitism is a key cause of mortality for *U. cardui* immatures [9]. In Europe, *U. cardui* larvae within Canada thistle galls are commonly parasitized by the koinobiont endoparasitoid *Eurytoma serratulae* Latreille (Hymenoptera: Eurytomidae) and the idiobiont ectoparasitoid *Eurytoma robusta* Mayr (Hymenoptera: Eurytomidae) [3,18,21], with the two species often co-occurring within a single gall [17].

Little is known about the insect species that occur within *U. cardui* galls in North Dakota, a north-central U.S. state, although the parasitoid community from nearby states has been studied [10]. Therefore, while monitoring the field releases of Canada thistle biocontrol agents [22,23], we collected additional *U. cardui* galls from Canada thistle in order to quantify the identity and density of insects associated with *U. cardui* galls and examine the relationships between gall size and insect density and biomass. We also provide the first report of a previously unknown predaceous beetle within the thistle galls, *Isohydnocera tabida* (LeConte) (Coleoptera: Cleridae).

## 2. Materials and Methods

### 2.1. Site Description and Sampling Methods

We sampled *U. cardui* galls on Canada thistle in 2012 and 2013 from field sites in the eastern and western parts of North Dakota, U.S. (Table 1). These sites were part of a state-wide Canada thistle biological control project, where in 2004, the North Dakota State Department of Agriculture released adult stem-mining weevils, *Hadroplontus* (*Ceutorhynchus*) *litura* Fabricius (Coleoptera: Curculionidae) (see Prischmann-Voldseth et al. [22,23] for details). In 2009, we established permanent transects at each site to enable the systematic collection of samples, with three 15.9 m-long transects extending out from the original weevil release site at the linear edge of the thistle patch, which was created by anthropogenic activity (e.g., a road or field edge), creating four inter-transect spaces [22].

In 2012, we collected *U. cardui* galls from May 2 to May 10, prior to the emergence of adult flies, in two to four of the inter-transect areas at each site (i.e., replicate) listed in Table 1. In each replicate, one person harvested galls for five minutes by clipping plant stems approximately 0.5–1.5 cm above and below the gall. Galls were placed in labeled self-sealing plastic bags and transported back to the lab in plastic coolers with ice packs. In 2013, we collected galls in the first week of May and focused on sites in Bowman and Stark counties where we had found *I. tabida* the previous year.

To assess insect emergence, within 48 h of sampling, we placed the galls in individual plastic cups (29.6 mL Solo^®^, Lake Forest, IL, USA) with muslin fabric covering a hole in the lid. The cups were maintained at room temperature (25 °C ± 5 °C) in the lab and monitored twice per week until an insect emerged, after which, the cups were checked every one to three days. In 2012, we monitored the cups until the end of June (11 d after the last insect emerged from a gall), after which, we considered emergence complete. In 2013, we monitored the galls daily after the first insect emergence event, and if no insects had emerged from a gall by the end of the observation period (i.e., 24 June 2013), the gall was dissected using a scalpel in order to obtain information on any remaining insects.

After collecting the insect data, we dried the galls to a constant mass in an oven at 49 °C and quantified their dry mass using a digital scale (Sartorius, model 1412, Westburg, NY, USA). We quantified the gall length (2a) and diameter along two axes at the widest point of the gall (2b,2c) using electric digital calipers (model 784EC, Sona Enterprises, Santa Fe Springs, CA, USA). We calculated the gall volume using the formula for an ellipsoid (*V* = 4/3π*abc*).

We placed the insects into labeled glass vials or plastic microtubes until they were identified, with the latter specimens stored in 70% ethanol only in 2013. We used White and Korneyev [1] to identify *U. cardui* and an online key [24] and other references to identify parasitoids [25,26]. We used Knull [27] and Optiz [28] to identify clerid beetles to species, which was verified by Dr. John Leavengood Jr. (john.m.leavengood@usda.gov). We deposited voucher specimens in the North Dakota State Insect Reference Collection, which is housed in the North Dakota State University Entomology department.

### 2.2. Statistical Analyses

Data were analyzed using JMP^®^ Pro 15.0.0 (SAS Institute Inc., Cary, NC, USA, 2019). Prior to analysis, we transformed the following variables to normalize their data distributions: SQRT (X + 0.5) for insect densities and log (X + 1) for gall and fly dry mass. We used a correlation analysis to test the associations among gall characteristics and *U. cardui* density and mass. The analyses included data from all viable galls, regardless of whether insects other than *U. cardui* were present, although correlations between gall characteristics and fly mass did not include data from galls where no *U. cardui* emerged. We used an ANOVA to examine the s of sample location on dependent variables, followed by a Tukey’s Honest Significant Difference post hoc test for mean separation. A non-parametric Kruskal–Wallis rank sum test, followed by a Steel–Dwass post hoc test for mean separation, i.e., a non-parametric test equivalent to Tukey’s Honest Significant Difference post hoc test [29,30], were used to analyze the differences in emerged wasp density between locations, with sites lacking wasps excluded from the analyses. Non-viable galls, or galls where no insects emerged or which, when dissected, lacked any living or dead insects, were not used in the analyses as these galls were likely more than a year old and all insects had previously emerged.

## 3. Results

*Urophora cardui* were widespread, and both males and females often emerged from the same gall (54% of galls with at least one fly in 2012), although only females (28%) and only males (17%) emerged from a sizeable percentage of galls. Across all sites, the mean (± SE) number of *U. cardui* adults that emerged from viable galls ranged from 1 to 16, with an average fly density of 3.26 ± 0.14 per gall in 2012 and 2.44 ± 0.12 in 2013 when using data from galls with at least one fly. In 2012, the location impacted the mean number of total *U. cardui* adults (both sexes) that emerged from the galls (*df* = _8262_, *F* = 6.89, *p* < 0.0001), as well as male (*df* = _8262_, *F* = 6.93, *p* < 0.0001), and female densities (*df* = _8262_, *F* = 2.80, *p* = 0.005; Table 2). The dry mass of all adult flies that emerged from the galls (i.e., total fly biomass per gall, 2012 data only) ranged from 0.8 to 43.2 mg and averaged 12.1 ± 0.6 mg (mean ± SE), with the highest fly biomass being from galls collected in Bowman and Griggs counties (*df* = _8262_, *F* = 10.17, *p* < 0.0001; Table 2). Using data from all the galls, the dry mass of an individual *U. cardui* adult averaged 3.6 ± 0.1 mg. When the data were limited to the galls where only a single *U. cardui* emerged, the average dry mass of one fly was 3.5 ± 0.2 mg for males (*n* = 31) and 3.4 ± 0.2 mg for females (*n* = 46).

**Figure 1:**
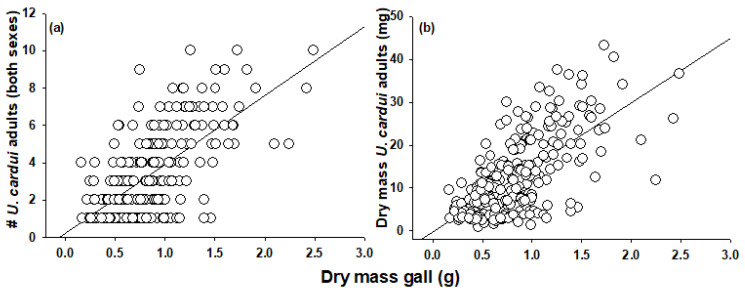
Correlations between (**a**) gall dry mass (g) and *U. cardui* density (male and female), R = 0.64, *p* < 0.0001 and (**b**) gall dry mass (g) and dry mass (mg) of all emerged adults, R = 0.63, *p* < 0.0001.

Gall volume and dry mass were highly positively correlated (2012, *R*^2^(359) = 0.90, *p* < 0.0001; 2013, *R*^2^(243) = 0.91, *p* < 0.0001; data not shown). In 2012, the gall dry mass and the number of *U. cardui* adults (both sexes) were positively correlated (*R*^2^(263) = 0.64, *p* < 0.0001; Figure 1a), as was gall dry mass and the dry mass of emerged *U. cardui* adults (*R*^2^(261) = 0.63, *p* < 0.0001; Figure 1b). The results were similar if data from galls housing insects other than *U. cardui* were excluded (data not shown).

**Figure 2:**
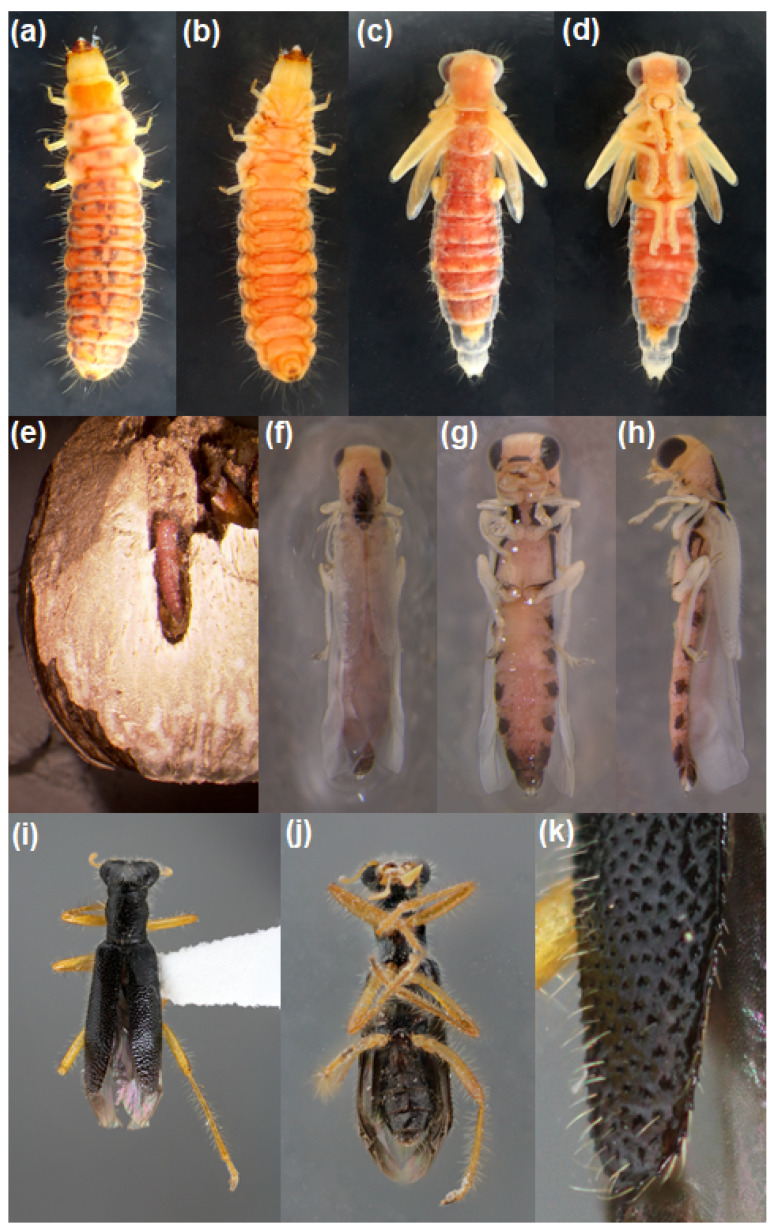
*Isohydnocera tabida*: (**a**) larva dorsal view, (**b**) larva ventral view, (**c**) pupa dorsal view, (**d**) pupa ventral view, (**e**) pupa within *U. cardui* puparial case in chamber of lignified gall on Canada thistle (**f**) teneral adult dorsal view, (**g**) teneral adult ventral view, (**h**) teneral adult lateral view, (**i**) adult dorsal view, (**j**) adult ventral view, (**k**), close-up of the tip of an elytron. Images (**a**–**d**,**i**–**k**) taken by G. Fauske, images (**e**–**h**) taken by S. J. Swenson and cropped by D. A. Prischmann-Voldseth.

During the gall dissections, we found the larvae, pupae, and adults of a clerid beetle species, *I. tabida*, inside *U. cardui* galls, as well as *I. tabida* pupae within *U. cardui* puparial cases (Figure 2), which appears to be a new host record. *Isohydnocera tabida* were only recovered from galls collected in the southwest corner of the state. When *I. tabida* were found, typically only one specimen was present per gall, although in 2013, two specimens were recovered from one gall, and in another case, three adults emerged from a single gall. In 2012, 11 specimens were recovered, and in 2013, when we focused our sampling efforts in that geographical area, we recovered 43 specimens from Stark and Bowman counties (Table 2).

**Figure 3:**
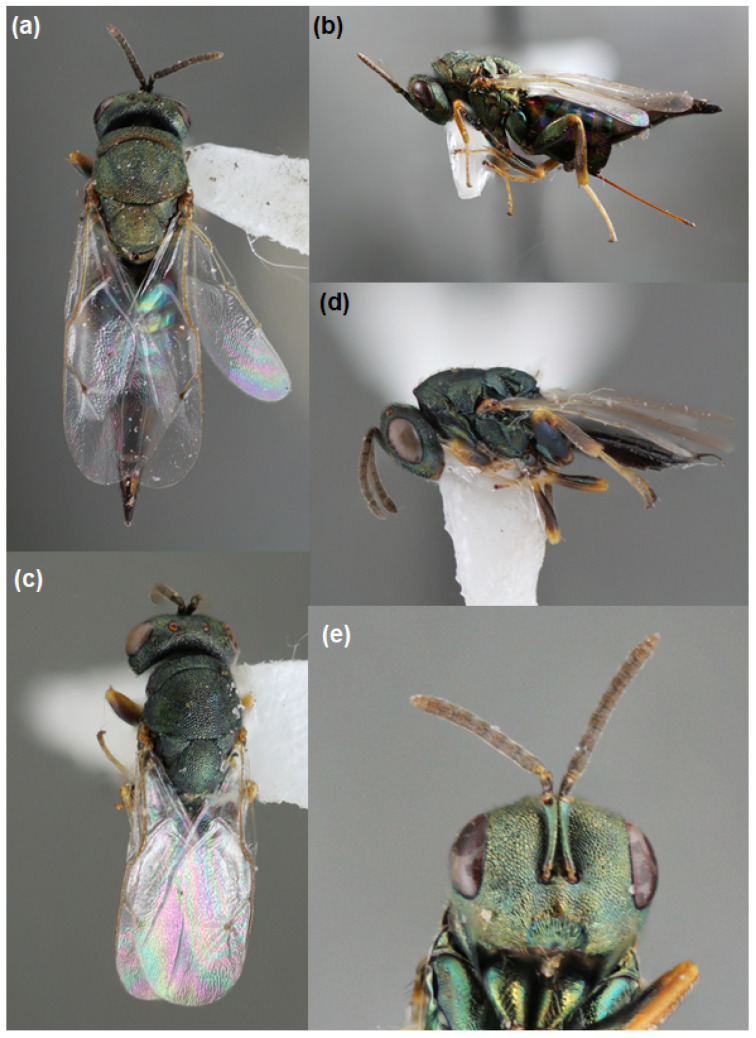
*Pteromalus elevatus*: (**a**) female dorsal view, (**b**) female lateral view, (**c**) male dorsal view, (**d**) male lateral view, (**e**) female close-up of head. Images taken by G. Fauske and cropped by D. A. Prischmann-Voldseth.

*Pteromalus elevatus* (Walker) (Hymenoptera: Pteromalidae) (syn. *Habrocytus elevatus*) was the most common parasitic wasp found (Figure 3), and male and female *P. elevatus* co-occurred within galls (Table 2). A few other parasitic wasp species were collected in the Ichneumonidae, Braconidae, and Chalcidoidea families, including what we believe was a *Schizoprymnus* sp. and a wingless wasp that was likely a *Eupelmus* (*Macroneura*) species, although the specimens could not be definitively identified and were extremely rare compared to *P. elevatus*. The number of individual parasitic wasps that emerged from a single gall ranged from 1 to 7, and when focusing on galls where at least one *P. elevatus* emerged, the density of adults per gall was similar among all sites (both sexes, Pearson’s χ^2^ = 7.17, df = 7, *P* = 0.412; males, Pearson’s χ^2^ = 6.93, *df* = 7, *P* = 0.436; females, Pearson’s χ^2^ = 0.83, *df* = 6, *P* = 0.991).

Most viable galls (81%) only housed a single species: *U. cardui* only (2012, 70.3%; 2013, 57.8%), parasitic wasp only (2012, 10.4%; 2013, 17.5%), and *I. tabida* only (2012, 1.0%; 2013, 5.6%). Two insects were occasionally (18%) associated with the same gall, which was primarily due to *U. cardui* co-inhabiting galls with wasps (2012, 15.9%; 2013, 12.2%), because *I. tabida* rarely co-occurred with *U. cardui* (2012, 1.0%; 2013, 4.7%) or parasitic wasps (2012, 0%; 2013, 1.2%). It was rare for all three insects to be present within a single gall (2012, 1.4%; 2013, 0.9%). Parasitic wasps typically emerged first, followed by *U. cardui*, with *I. tabida* emerging throughout the observation period. Occasionally we recovered other arthropods from the galls, such as spiders or minute brown scavenger beetles (Lathridiidae), which were likely transiently associated with the galls.

## 4. Discussion

Here, we report the first occurrence of *Isohydnocera tabida* reared from *U. cardui* galls on Canada thistle. Our observation of *I. tabida* immatures inside the galls and a pupa inside the puparium of *U. cardui* suggests that the clerids are preying on the gallflies. Clerid beetles, also known as checkered beetles, are typically brightly colored and patterned as adults and prey on beetles and other insects inhabiting wood, although some have been reported to eat pollen, grasshopper eggs, and immature Lepidoptera and Hymenoptera [28,31,32,33], while Morrill et al. [34] have reported adult and larval *Phyllobaenus dubius* (Wolcott) preying on wheat stem sawfly larvae, *Cephus cinctus* Norton (Hymenoptera: Cephidae), within wheat stems.

*Isohydnocera* was previously within the genus *Hydnocera*, with insects in the latter genus having been reported to feed on cryptic insect larvae in multiple orders, including wood-borers, stem-miners, and gall-makers [31,32,35]. Within *Isohydnocera*, *I. curtipennis* is one of the best-known species. In addition to consuming wood-boring insects [36], *I. curtipennis* adults were reared from *Euura salicis-nodus* Walsh (Hymenoptera: Tenthredinidae) galls on willow (Salicales: Salicaceae) trees in Fort Collins, Colorado [37] and *Gnorimoschema gallaesolidaginis* Riley (Lepidoptera: Gelechiidae) galls on goldenrod (Asterales: Asteraceae, *Solidago* sp.) plants [32]. In the latter case, Sabrosky [32] concluded that an *I. curtipennis* larva had parasitized a single *G. gallaesolidaginis* pupa from a plant collected near Medora, Kansas, and noted that the adult beetle emerged from the gall in May through a single round exit hole.

De Smet-Moens [38] reported occasionally collecting an unidentified clerid beetle larva in the genus *Phyllobaenus* or *Isohydnocera* feeding on *Orellia ruficauda* (F.) (Diptera: Tephritidae) on Canada thistle in southern Montana. Price [10] reared four different species of clerid beetles from *U. cardui* galls on Canada thistle and indicated that they were predators: *Dolichosoma foveicolle* (Kirby) from Oregon and Washington, *Enoclerus rosmarus* (Say) from South Dakota, *P. humeralis* (Say) from North Dakota (Standing Rock Indian Reservation, N 46°13′47.1″ W 100°42′40.4″), and *I. curtipennis* (Newman) from Montana.

*Isohydnocera tabida* has been reported in multiple locations and habitats in Canada and the central and eastern U.S., and appears to have a widespread distribution. Searching for “*Isohydnocera tabida*” in SCAN (Symbiota Collections of Arthropods Network, scan-bugs.org) [39], which has specimen data from over 225 North American arthropod specimen and observation collections, resulted in 10 images of adults and 172 specimen records from two Canadian provinces (Québec and Ontario), and sixteen U.S. states. We found similar results when we searched the Global Biodiversity Information Facility open access database [40,41]. The NDSIRC (North Dakota State Insect Reference Collection, NDSU, Fargo ND) has 15 specimens of *I. tabida* adults from North Dakota, South Dakota, and South Carolina, including some islands in the latter state (Table 3). With regard to the literature records, Chapin [42] indicates that *I. tabida* is found along the eastern coast of the U.S., into Canada and west to Kansas and Nebraska, and Wolcott [43] reports the presence of *I. tabida* in Ontario, Canada and five U.S. states: Alaska, Illinois, Indiana, Kansas, and Wisconsin. *Isohydnocera tabida* has been collected from the salt marshes in North Carolina [44] and from three of the Boston Harbor Islands (i.e., Great Brewster, Middle Brewster, and Ragged) off the Massachusetts coast [45].

Specimen records appear to be for adults, which have been collected from April (Texas) to August (Massachusetts, Wisconsin), and are most frequently collected in June and July. The records indicate that adults were collected by sweeping or beating vegetation (e.g., ferns, grasses, weeds, and herbaceous plants) in meadows or in fragmented and disturbed landscapes (NDSIRC records, [27,39,42,46]). Mawdsley [47] suggested that *I. tabida* was likely found in prairie habitats, and in Iowa, multiple adults were collected from lead plants (Fabales: Fabaceae, *Amorpha canescens* Pursh) at Rahel’s Prairie within the Hitchcock Nature Center [48]. In Wisconsin, adults were knocked off black locust branches (Fabales: Fabaceae, *Robinia pseudoacacia*) or hand collected from a walnut tree (Fagales: Juglandaceae, *Juglans* sp.) [46]. One adult was collected from milkweed (Gentianales: Apocynaceae, *Asclepias syriaca* L.) in Bowling Green, Ohio [49]. Adults have also been collected using Malaise and light traps [39].

Even though multiple *I. tabida* adults have been collected, there are virtually no records or information on the life history of the immatures. Böving and Champlain [31] and Böving and Craighead [50] present identification keys for subfamily or genus and illustrations of larval Cleridae, although information on *I. tabida* is lacking. Knull [27] indicated that *I. tabida* was found in plant stems infested with tumbling flower beetle larvae (Coleoptera: Mordellidae), which likely refers to the reports from East Falls Church, Virginia, where pupae were found in annual plant stems infested with mordellid larvae [31] and from Staten Island, New York, where Leng and Davis [51] indicated that *I. tabida* preyed on mordellid larvae in the stems of annual plants in June. Our discovery that *I. tabida* larvae and pupae occur within *U. cardui* galls on Canada thistle could explain both the lack of information on immatures and the widespread distribution of adults, as this weed has a cosmopolitan distribution. However, the clerid could also be preying on parasitoid wasps within the galls. Larvae of a related species (*Phyllobaenus*) have been observed consuming *Microbracon* (Hymenoptera: Braconidae) and *Eurytoma* pupae within the galls of the moth *Gnorimoschema baccharisella* Busck (Lepidoptera: Gelechiidae) on coyote brush plants (Asteraceae: *Baccharis pilularis*) [52].

Information on *U. cardui* predators is scarce, although some moth larvae, e.g., *Metzneria* spp. (Lepidoptera: Gelechiidae), *Homoeosoma* spp. (Lepidoptera: Pyralidae), and *Eucosma* spp. (Lepidoptera: Tortricidae) have been reported to prey on larvae of other *Urophora* species [17]. Insect-induced plant galls are also vulnerable to predation by vertebrates such as mice and birds [53], with higher predation rates on large galls [54], although Freese and Zwölfer [14] indicated that predation of *U. cardui* galls does not depend on gall size. Parasitoids are considered to be the primary cause of mortality for larval *U. cardui* [9,17].

In the Palearctic region, *Eurytoma robusta* and *E. serratulae* are the most common hymenopteran parasitoids of *U. cardui* larvae, and the two species can co-occur within a single gall [3,21]. In contrast, the ectoparasitoid *P. elevatus* was the dominant parasitoid species in our study. *Pteromalus elevatus* primarily parasitizes tephritid flies that feed on plants within the Asteraceae family [55,56,57]. This species is associated with *U. cardui* throughout Europe, where it is generally considered a minor parasitoid [11,58,59]. In southern Finland, *P. elevatus* was present at 25 out of 75 sites [58], and in northern Germany and Denmark, Johannesen and Seitz [18] found *P. elevatus* parasitoids at 13 of the 20 sites they sampled, and its rate of parasitizing *U. cardui* ranged up to 11%. However, the general conclusion is that in the Palearctic region, *P. elevatus* does not have a major impact on *U. cardui* populations [9,17,21].

In the Nearctic region, *U. cardui* were released in Canada to control Canada thistle starting in 1974 [6], and by 1978–1979, 3% of fly larvae were parasitized by *P. elevatus*. Similar to our observations, Peschken and Derby [20] found *P. elevatus* was the dominant parasitoid of *U. cardui* in New Brunswick, Canada, and did not find any *E. robusta* or *E. serratulae*. They indicated that *P. elevatus* was accidentally introduced to North America (Newfoundland, Canada), and suggested that the levels were too low to negatively impact *U. cardui* [20]. Parasitoids associated with *U. cardui* were surveyed in the western U.S. (Washington, Oregon, Idaho = 13 sites; Montana = 6 sites), and the Midwest (North Dakota = 1 site; South Dakota = 15 sites) [10]. Price [10] reared 11 parasitoid wasps from *U. cardui* galls, and *P. elevatus* was the dominant parasitoid associated with *U. cardui* at all their study sites, although they reported a low rate of parasitism (i.e., 11.4% of galls) and concluded that parasitoids did not limit the control efficacy of *U. cardui*. A previous survey of insects associated with Canada thistle in Montana did not list *U. cardui*, and the authors indicated that this species had not established after its initial release in 1978 [38,60].

We reared a small number of uncommon parasitic wasp species from *U. cardui* galls, including an unidentified species of *Schizoprymnus*. Price (2014) rarely recovered *Schizoprymnus* from *U. cardui* galls and did not find any in North Dakota [10]. Wasps in this genus typically parasitize beetle larvae but are also known from dipteran and lepidopteran hosts [26], so they may have been parasitizing either *U. cardui* or *I. tabida*, although we did not find the latter and this wasp species at the same location.

The average density of *U. cardui* per gall at our study sites was similar to that in other areas, including the Midwest and western U.S. (1.16 ± 0.14 SE adult *U. cardui* emerged per gall) [10], Denmark and northern Germany (3.6 ± 1.7 SD cells per gall, ranging from 1 to 12) [18], eastern France (3.86 ± 2.43 cells per gall, ranging from 1 to 23) [14], central Europe (3.38 ± 0.66 cells per gall with a range of means from 2.29 to 4.91) [14], and Canada (5.8 ± 3.7 SD cells per gall, ranging from 1 to 17) [16]. We found that gall size was positively related to *U. cardui* density, and others have also documented positive correlations between gall size and the number of larval chambers within the gall and the number of *U. cardui* larvae and emerged adults [6,10,16,18,61]. Freese and Zwölfer [14] found that the more *U. cardui* larvae within a gall, the lower the larval mortality, the higher the larval mass, and the higher the fitness of the resulting adults.

Exposure to air is believed to trigger *U. cardui* pupation [8,11], which typically occurs in the spring when galls become wet and the callus tissue blocking the larval tunnels degrades [13]. Because moisture facilitates pupation and adult emergence [13], and we placed detached galls in plastic cups without supplemental moisture, our insect densities are likely conservative, which is supported by our observations of low levels of live and dead larvae, pupae, and adults in dissected galls. We found little to no evidence of *U. cardui* death due to pathogens.

In conclusion, *P. elevatus* was the primary parasitoid of *U. cardui* on Canada thistle across North Dakota, with certain sites having higher parasitoid densities than other areas. *Isohydnocera tabida* appeared to be geographically restricted to the SW corner of the state, and our observations suggest that this species preys on *U. cardui* and that their immatures use the galls as protected overwintering sites, as has been suggested for other clerid beetle species [35]. *Isohydnocera tabida* may also opportunistically feed on parasitoids, as evidenced by the fact that clerid beetles rarely co-occurred with other insects within a gall.

## Figures and Tables

**Table 1:** Location and environmental characteristics of collection sites and sampling dates.

Site ^1^	ND ^2^ County	Latitude N, Longitude W (Decimal Degrees)	NDAWN ^3^ Weather Station	Avg. Air Temp. (°C)		Avg. Precipitation (cm)		Date Galls Collected ^4^
				May	Yearly	May	Yearly	
Tr_a	Trail	47.577, 96.859	Hillsboro ND	13.3	5	6.86	54.99	4 May 2012
Tr_b	Trail	47.312904, 97.409602	Mayville ND	12.7	4.4	6.83	54.79	4 May 2012
Ra	Ransom	46.350056, 97.933539	Lisbon ND	13.9	6.1	7.49	53.75	2 May 2012
Gr	Griggs	47.514863, 98.366467	Cooperstown ND	12.2	3.9	7.42	54.89	4 May 2012
St_a	Stark	46.848512, 102.892616	Dickinson ND	11.7	5.6	5.89	42.49	10 May 2012 20 May 2013
St_b	Stark	46.849122, 102.892973	Dickinson ND	11.7	5.6	5.89	42.49	
St_c	Stark	46.854804, 102.893682	Dickinson ND	11.7	5.6	5.89	42.49	
Sp	Slope	46.294698, 103.634989	Bowman ND	11.7	6.1	6.27	39.65	11 May 2012
Bo	Bowman	46.223459, 103.702719	Bowman ND	11.7	6.1	6.27	39.65	11 May 2012 21 May 2013

^1^ Sites are arranged from east to west; the three sites in Stark county were considered unique when they were established, but are adjacent to one another, and so we sampled them collectively in 2013. ^2^ ND = North Dakota, U.S. ^3^ Climate data from NDAWN weather stations (North Dakota Agricultural Weather Network, https://ndawn.ndsu.nodak.edu accessed on 13 July 2022). ^4^ Galls from St_a, St_b, St_c collected on 10 May 2012 and 20 May 2013; galls from Bo collected on 11 May 2012 and 21 May 2013.

**Table 2:** Data on insects associated with galls collected in 2012 and 2013.

Site ^1^	# Galls ^2^					# Adult Flies Per Gall (Mean ± SE) ^3^			Fly Biomass Per Gall (Mean ± SE mg dry wt.) ^3^	# *P. elevatus* Per Gall (Mean ± SE) ^4^		
	C	V	+I	+U	+P	M and F	M	F	M and F	M and F	M	F
2012												
Tr_a	8	8	0	8	0	2.13 ± 0.52b	1.00 ± 0.42ab	1.13 ± 0.35ab	6.36 ± 1.49b	n/a	n/a	n/a
Tr_b	54	37	0	34	9	2.59 ± 0.33b	1.44 ± 0.22ab	1.15 ± 0.19b	8.30 ± 1.09b	1.64 ± 0.20a	1.25 ± 0.16a	1.20 ± 0.20a
Ra	41	12	0	11	1	2.09 ± 0.39b	0.46 ± 0.16b	1.64 ± 0.34ab	5.92 ± 1.08b	4.00 ± ^5^a	1.00 ± ^5^a	n/a
Gr	38	37	0	36	1	4.64 ± 0.37a	2.36 ± 0.26a	2.28 ± 0.24a	18.23 ± 1.49a	2.00 ± ^5^a	1.00 ± ^5^a	1.00 ± ^5^a
St-a	62	59	5	51	25	3.02 ± 0.34b	1.29 ± 0.19b	1.73 ± 0.24ab	10.84 ± 1.27b	1.81 ± 0.23a	1.32 ± 0.19a	1.31 ± 0.15a
St-b	34	27	0	24	8	3.04 ± 0.41ab	1.75 ± 0.27ab	1.29 ± 0.22ab	10.54 ± 1.42b	1.75 ± 0.25a	1.13 ± 0.13a	1.25 ± 0.25a
St-c	62	51	5	41	21	2.41 ± 0.25b	0.81 ± 0.15b	1.61 ± 0.22ab	8.64 ± 0.90b	1.86 ± 0.26a	1.35 ± 0.17a	1.23 ± 0.12a
Sp	25	22	0	17	9	2.59 ± 0.49b	1.44 ± 0.22ab	1.24 ± 0.27ab	9.51 ± 2.01b	2.56 ± 0.50a	1.78 ± 0.32a	1.17 ± 0.17a
Bo	44	41	1	41	3	4.66 ± 0.37a	2.51 ± 0.30a	2.15 ± 0.22a	19.31 ± 1.58a	1.00 ± 0.00a	1.00 ± 0.00a	1.00 ± ^5^a
2013												
St-a-c	480	124	17	82	37	1.83 ± 0.15	n/a	n/a	n/a	1.71 ± 0.23	n/a	n/a
Bo	211	196	23	160	65	2.74 ± 0.15	n/a	n/a	n/a	1.92 ± 0.17	n/a	n/a

^1^ Sites are arranged from east to west; the three sites in Stark county were considered unique when they were established, but are adjacent to one another, and so we sampled them collectively in 2013. ^2^ C = galls collected, V = galls with at least one insect, +I = galls with at least one *I. tabida*, +U = galls with at least one *U. cardui*, +P = galls with at least one *P. elevatus.* ^3^ Data from galls with at least one *U. cardui*, M = males, F = females. ^4^ Data from galls with at least one *P. elevatus*, M = males, F = females. ^5^ Insects only recovered from one gall, so there’s no variance, within a column values sharing the same letter are not significantly different at alpha = 0.05.

**Table 3:** List of *I. tabida* specimens (pinned adults) in the NDSIRC (North Dakota State Insect Reference Collection, NDSU, Fargo, ND, U.S.).

State ^1^	County	Location	Date	Year	Collector/Identifier	Other Label Data ^3^
ND	Barnes	S. of V[alley] city	6-vi	1963	D.[G.] Aarhus	-
ND	Ransom	-	6-vi	1963	R.L. Post/D.E. Foster	-
ND	Sargent	Tewaukon NWR ^2^	4-vi	1964	D.G. Aarhus	-
SD	Brookings	Brookings	28-vi	1965	V.M. Kirk	S[weeping], 2 spp.
SD	Brookings	White	18 vii	1966	V.M. Kirk	-
SD	Union	Elk Point	20-vi	1968	V.M. Kirk/W.F. Barr	-
SC	Charleston	Kiawah	10-v	1963	V.M. Kirk	2 spp.
SC	Charleston	Seabrook	8-x	1962	V.M. Kirk/[J.N.] Knull	-
SC	Charleston/Colleton	Edisto Is.	9-x	1962	V.M. Kirk	Sweeping beach grass, 2 spp.
SC	Georgetown	Pawley’s Is.	7-viii	1962	V.M. Kirk	S[weeping], 2 spp. ‘159’ [?]
SC	Sumter	Sumter	15-vii	1964	V.M. Kirk	-

^1^ ND = North Dakota, SD = South Dakota, SC = South Carolina. ^2^ NWR = National Wildlife Refuge. ^3^ 2 spp. indicates one male and one female.

## Data Availability

The data presented in this study are available on request from the corresponding author.
